# Association of Blood-Based Biomarkers and 6-Month Patient-Reported Outcomes in Patients With Mild TBI: A CENTER-TBI Analysis

**DOI:** 10.1212/WNL.0000000000210040

**Published:** 2024-12-09

**Authors:** Daniel P. Whitehouse, Lindsay Wilson, Endre Czeiter, Andras Buki, Kevin K.W. Wang, Nicole von Steinbüchel, Marina Zeldovich, Ewout Steyerberg, Andrew I.R. Maas, David K. Menon, Virginia F.J. Newcombe, for the CENTER-TBI Investigators and Participants

**Affiliations:** From the Perioperative, Acute, Critical Care and Emergency Medicine (PACE) (D.P.W., D.M., V.F.J.N.), Department of Medicine, University of Cambridge, Addenbrooke's Hospital; Division of Psychology (L.W.), University of Stirling, United Kingdom; Department of Neurosurgery (E.C.), Medical School, and Neurotrauma Research Group (E.C.), Szentagothai Research Centre, University of Pecs, Hungary; Department of Neurosurgery (A.B.), Faculty of Medicine and Health, Örebro University, Sweden; Department of Neurobiology (K.K.W.W.), Center for Neurotrauma, Multiomics & Biomarkers (CNMB) Neuroscience Institute, Morehouse School of Medicine (MSM), Atlanta, GA; Program for Neurotrauma, Neuroproteomics and Biomarker Research (K.K.W.W.), Departments of Emergency Medicine, Psychiatry and Neuroscience, University of Florida, McKnight Brain Institute, Gainesville; Institute of Psychology (N.v.S., M.Z.), University of Innsbruck; Faculty of Psychotherapy Science (M.Z.), Sigmund Freud University, Vienna, Austria; Department of Biomedical Data Sciences (E.S.), Leiden University Medical Center, the Netherlands; Department of Neurosurgery (A.I.R.M.), Antwerp University Hospital, Edegem; and Department of Translational Neuroscience (A.I.R.M.), Faculty of Medicine and Health Science, University of Antwerp, Belgium.

## Abstract

**Background and Objectives:**

There is seemingly contradictory evidence concerning relationships between day-of-injury biomarkers and outcomes after mild traumatic brain injury (mTBI). To address this issue, we examined the association between a panel of biomarkers and multidimensional TBI outcomes.

**Methods:**

Participants with mTBI (Glasgow coma scores [GCSs] 13–15) were selected from Collaborative European NeuroTrauma Effectiveness Research in Traumatic Brain Injury, a European observational study recruiting patients with TBI with indication for brain CT and presentation within 24 hours. Exclusion criteria for this secondary analysis were age younger than 16 years, incomplete biomarker panel, death, or no recorded outcomes. Participants were separated into 2 groups, CT-negative and CT-positive. Multivariable binary logistic regression was used to assess the relation between the log biomarker level (glial fibrillary acidic protein [GFAP], neurofilament light [NfL], neuron-specific enolase [NSE], S100 calcium-binding protein B [S100B], tau, ubiquitin C-terminal hydrolase L1 [UCH-L1]) and dichotomized 6-month outcomes (functional outcomes [GOSE score <8], health-related quality of life [HRQoL; Quality of Life after Brain Injury-Overall Scale (QOLIBRI-OS) score <52, Short-Form 12-Item Survey version 2 Mental Component Summary (SF12v2 MCS) score <40, Short-Form 12-Item Survey version 2 Physical Component Summary (SF12v2 PCS) score <40], persistent postconcussion symptoms [Rivermead Post-Concussion Symptoms Questionnaire score ≥16], anxiety disorder [Generalized Anxiety Disorder-7 (GAD-7) score ≥8], depression [Patient Health Questionnaire-9 (PHQ-9) score ≥10], and post-traumatic stress disorder [PTSD Checklist for DSM-5 (PCL-5) score ≥33]).

**Results:**

A total of 1,589 participants (865 CT-negative, 724 CT-positive) were included (77% GCS 15, median age 52 years, 66% male). Higher biomarker levels were associated with a GOSE score <8: CT-negative: S100B (odds ratio [OR] 1.78, 95% CI 1.43–2.23) and UCH-L1 (OR 1.16, 95% CI 1.01–1.33); CT-positive: GFAP (OR 1.22, 95% CI 1.11–1.36), NfL (OR 1.30, 95% CI 1.11–1.52), S100B (OR 1.51, 95% CI 1.23–1.86), tau (OR 1.36, 95% CI 1.17–1.59), and UCH-L1 (OR 1.34, 95% CI 1.17–1.53). In CT-positive participants, positive association was seen between NfL (OR 1.3, 95% CI 1.06–1.60) and UCH-L1 (OR 1.28, 95% CI 1.07–1.54) with QOLIBRI-OS; S100B (OR 1.32, 95% CI 1.02–1.70) with SF12v2 PCS; and NSE (OR 1.52, 95% CI 1.06–2.18) and UCH-L1 (OR 1.21, 95% CI 1.01–1.46) with the GAD-7. However, in CT-negative participants only, negative associations were seen between GFAP and impairment on the QOLIBRI-OS (OR 0.76, 95% CI 0.66–0.88), SF12v2 MCS (OR 0.71, 95% CI 0.61–0.82), SF12v2 PCS (OR 0.79, 95% CI 0.68–0.91), GAD-7 (OR 0.80, 0.68–0.95), PHQ-9 (OR 0.80, 95% CI 0.68–0.93), and PCL-5 (OR 0.80, 95% CI 0.66–0.97).

**Discussion:**

Participants with higher biomarker levels had greater odds of impaired functional recovery. However, in CT-negative participants, higher GFAP concentrations were associated with better HRQoL and less impaired mental health. Further exploration is required of the patient phenotypes that may explain the relationships observed in this analysis.

## Introduction

After a mild traumatic brain injury (mTBI), around 50% of patients report functional impairment a year after injury.^1^ Furthermore, associations are found between a history of mTBI and the development of psychiatric illness^2^ and impairments in health-related quality of life (HRQoL).^3,4^ Although there have been efforts to develop prognostic models for patient HRQoL outcomes, the complexity of the relationship between patient, injury, and outcome has resulted in only moderate predictive performance.^5^

A CT head scan remains the primary investigation in mTBI, yet only a minority of patients have traumatic lesions.^6^ While the presence and pattern of lesions (CT-positive or complicated mTBI) is associated with worse functional and HRQoL outcomes compared with patients with no lesions (CT-negative or uncomplicated TBI),^7^ even those patients without any visible lesions may have poor outcomes.^1^ It is clear that CT findings do not provide a complete picture of the extent of injury sustained after mTBI.^8^ Blood biomarkers offer a way to further characterize the intracranial injury burden,^8,9^ with much of the current research focusing on a panel of proteomic biomakers including the astroglial markers glial fibrillary acidic protein (GFAP) and S100 calcium-binding protein B (S100B), the axonal marker neurofilament light (NfL), the dendritic marker tau, and the neuronal markers neuron-specific enolase (NSE) and ubiquitin C-terminal hydrolase L1 (UCH-L1).^10^

The acute concentrations of this panel of biomarkers are associated with the overall functional outcome after mTBI and add value in prognostic modeling with higher levels associated with worse functional outcomes.^11,12^ By contrast, however, lower concentrations of GFAP, UCH-L1, and S100B have been described in patients with greater levels of postconcussive symptoms while lower GFAP concentrations have been associated with greater probability of post-traumatic stress disorder (PTSD).^13,14^ These apparently paradoxical findings may indicate potentially discordant biological processes, with factors other than injury severity driving the particular outcomes. This issue is important because evidence concerning the origin of symptoms reported after TBI may influence the management of patients and help to inform the design of clinical trials.

We aimed to assess the association between day-of-injury biomarkers and 6-month outcomes, including mental health and HRQoL, in participants of the Collaborative European NeuroTrauma Effectiveness Research in Traumatic Brain Injury (CENTER-TBI) study after CT-negative or CT-positive mTBI.^15,16^ The primary hypothesis of the analysis is that an increased severity of intracranial injury, as indicated by the level of day-of-injury biomarkers, will be positively associated with worse patient-reported outcomes in both patients with CT-negative and CT-positive mTBI.

## Methods

Participants were selected from the CENTER-TBI core study (EC grant 602150), recruited from 65 clinical sites across 18 countries in Europe and Israel between December 19, 2014, and December 17, 2017. The inclusion criteria for CENTER-TBI were presentation within 24 hours of head injury, with an indication for CT brain imaging according to local guidelines.^15,16^ Participants were included in this secondary analysis if they had a baseline Glasgow coma score (GCS) between 13 and 15 and were aged older than 16 years, had at least 1 outcome of interest at 6 months, survived to 6 months, and had a complete panel of biomarkers and CT imaging within 24 hours of injury. Participants were stratified into 2 groups, CT-negative and CT-positive, depending on the presence or absence of any traumatic intracranial findings on acute CT (not including skull fracture).

### Procedures

Clinical variables were collected using the Quesgen e-CRF (Quesgen Systems Inc., Burlingame, CA) and hosted on the International Neuroinformatics Coordinating Facility (INCF; Karolinska Institutet, Stockholm, Sweden) and extracted using the INCF Neurobot tool (data version 3.0; INCF). Radiologic findings were obtained from centrally performed reading of the acute CT scan in accordance with the Common Data Elements scheme for TBI.^17^

Blood samples were obtained within 24 hours from injury, centrifuged within 60 minutes, and stored locally at −80° before shipment to the CENTER-TBI biobank (Pecs, Hungary).^10^ Biomarker analysis was performed at Pécs, Hungary, and Gainesville, FL. The Single-Molecule Arrays (SiMoA)–based Human Neurology 4-Plex B assay was used to analyze GFAP, NfL, total tau, and UCH-L1 on the SR-X benchtop assay platform (Quanterix Corp., Lexington, MA) while NSE and S100B were analyzed on the e602 module of the cobas 8000 modular analyzer (Roche Diagnostics, Mannheim, Germany) using an electrochemiluminescence immunoassay kit (Elecsys S100 and NSE assays).

### Outcomes

Outcomes were assessed at 6-month follow-up. The Glasgow Outcome Scale Extended (GOSE), assessed by either a structured interview or a questionnaire completed by the patient or carer, was used to rate functional outcomes.^18^ Assessments were scored centrally, and if there was no value recorded in the per-protocol time window (5–8 months), an imputed 180-day GOSE was derived when data were available outside this range.^19^ The GOSE scores was dichotomized to complete (GOSE score 8) and incomplete (GOSE score <8) recovery. In addition, a set of 5 questionnaire assessments were completed by the patient. Disease-specific HRQoL was measured using the Quality of Life after Brain Injury-Overall Scale (QOLIBRI-OS).^20,21^ The total QOLIBRI-OS score was dichotomized, with a score less than 52 indicating impaired HRQoL. Physical and mental generic HRQoL was assessed using the physical (PCS) and mental (MCS) component summary scores from the SF12v2.^22^ Where the PCS or MCS scores from the SF-12v2 were missing, a score was derived from the corresponding items on the SF-36v2. A cutoff of 40 was considered impaired in reference to both the MCS and PCS. Persistent postconcussion symptoms (PPCSs) were examined using the Rivermead Post-Concussion Symptoms Questionnaire (RPQ), with a total score of 16 or greater considered as PPCS.^14,23^ Anxiety was assessed using the Generalized Anxiety Disorder-7 (GAD-7) questionnaire with a score of 8 or greater indicating probable anxiety disorder.^24^ Depression symptoms were measured using the Patient Health Questionnaire-9 (PHQ-9) with a score of 10 or greater indicating likely depression.^25^ PTSD was evaluated using the PTSD Checklist for DSM-5 (PCL-5) with a score of 33 or greater indicating probable PTSD.^26^

### Statistical Analysis

Participants' sociodemographic and injury characteristics are presented as medians (interquartile range) for continuous variables and number (percentage) for categorical variables. The biomarker concentrations and the occurrence of dichotomized outcomes were compared between CT-negative and CT-positive participants using the Wilcoxon test for continuous variables and the χ^2^ test for categorical variables.

In participants with a complete outcome profile, the copresentation of outcomes is presented with an upset plot. Associations between the dichotomized outcomes were assessed using phi with adjustment for the maximum value.^27^ Principal component analysis (PCA) was used to identify potential relationships between the dichotomized outcome variables. The binary outcome variables were inputted into the PCA algorithm, and a scree plot was used to determine the number of principal components using the elbow method. A loading plot with no rotation was created to explore the covariance of the outcome variables.

Biomarker concentrations were compared between participants with and without the dichotomized outcome of interest using a Wilcoxon test and adjusted for multiple comparisons using the Benjamini-Hochberg method.^28^

The association between biomarkers and outcomes was further compared using binary logistic regression to allow for adjustment for potential confounders. Covariates were selected based on review of previous literature and included patient age at time of injury, sex, extracranial injury, and time to biomarker sampling.^29-32^ The degree of extracranial injury was calculated using a total abbreviated injury scale (AIS), including cervical spine injury, but with head and neck, and brain injury AIS removed. The linearity assumption was assessed using scatter plots with multicollinearity assessed with the variance inflation factor (VIF). All VIFs were below 2, indicating no significant collinearity. Biomarker concentrations were log transformed in all models to meet the linearity in the logit assumption. Results are presented as adjusted odds ratios (ORs), Nagelkerke *R*^2^, Akaike information criterion, 95% CIs, and significance values.

Analysis was performed using R (version 4.2.2) in RStudio.

### Missing Data

Participants were only included with a recording of 1 or more outcome variables. There were no missing covariate data in the logistic regression analysis, so a complete case analysis is presented for each outcome variable.

### Sensitivity Analyses

Post hoc sensitivity analyses examined the influence on the primary results of the inclusion of participants' pre-existing psychiatric illness (any medical history of anxiety, depression, sleep disorder, schizophrenia, substance abuse, or other psychiatric diagnoses) as an additional covariate in the regression models and the impact of only including participants meeting a stricter mTBI case definition in keeping with the American Congress of Rehabilitation Medicine (ACRM) diagnostic criteria for mTBI (GCS <15, post-traumatic amnesia, or a proven loss of consciousness) as a targeted subanalysis.^33^ The analyses were limited to CT-negative participants and concentrated on the GFAP associations, owing to the relationships observed in the primary results. To evaluate the impact of missing outcome data biasing the observed contrasts between outcome measures, especially in reference to the GOSE that had a greater data availability compared with the other measures, the primary analysis was repeated using a subset of participants who had complete data for all outcome measures.

### Standard Protocol Approvals, Registrations, and Patient Consents

The CENTER-TBI study (EC grant 602150) has been conducted in accordance with all relevant laws of the EU if directly applicable or of direct effect and all relevant laws of the country of the recruiting site.^34^ Informed consent was obtained for all recruited participants from the patients and/or the legal representative/next of kin. A full list of recruitment sites, ethical committees, approval numbers, and approval dates can be found online.^34^

### Data Availability

Data-sharing requests are conditional on approved study proposal with no end dates to the availability, with further details provided online.^35^

## Results

### Demographics

**Figure 1:**
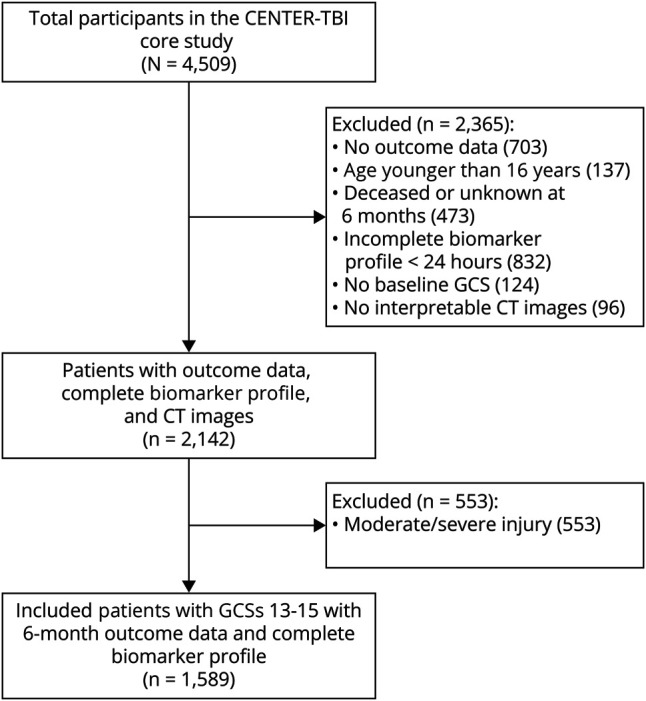
Flowchart of the Derived Sample CENTER-TBI = Collaborative European NeuroTrauma Effectiveness Research in Traumatic Brain Injury; GCS = Glasgow coma score.

**Figure 2:**
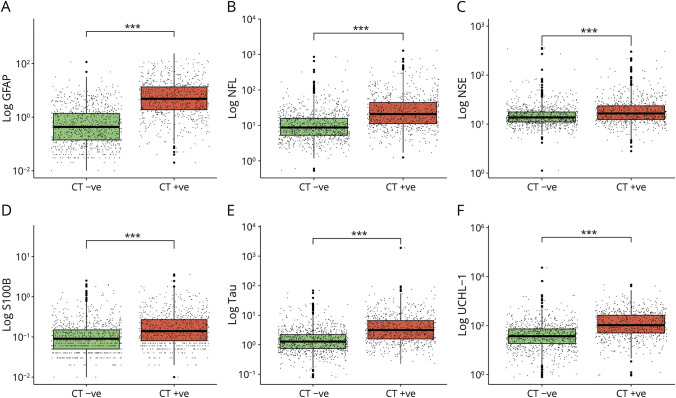
Distribution of Biomarker Concentration Between Participants With CT-Negative and CT-Positive Mild TBI Log10 of day-of-injury biomarker concentration in participants with mild TBI divided by presence of acute CT abnormality. Significance derived from the Wilcoxon test with adjustment for multiple comparisons with the Benjamini-Hochberg method. ***p ≤ 0.001, **p < 0.01, *p < 0.05 (Table 1 for details). GFAP = glial fibrillary acidic protein; NfL = neurofilament light; NSE = neuron-specific enolase; S100B = S100 calcium-binding protein B; TBI = traumatic brain injury; UCHL1 = ubiquitin C-terminal hydrolase L1.

A total of 1,589 participants were included in this analysis (Figure 1), 865 CT-negative and 724 CT-positive, with a median age of 52 years, 66% male, 96% White, and 77% with GCS 15. 47% were injured in a fall, and 37% were injured in a road traffic incident (Table 1). Missing outcome data in the cohort ranged from 0% to 29% (eFigure 1, eTable 1). The concentration of all biomarkers was significantly raised in participants with CT-positive mTBI compared with CT-negative (Table 1, Figure 2). The time to biomarker sampling in reference to the patient group and outcomes is displayed in the supplement (eTable 2, eFigure 2).

**Table 1:** Demographics of the Patient Population Included in This Analysis and a Comparison Between CT-Negative and CT-Positive Participants

Demographics	All participants (N = 1,589)	CT-negative (N = 865)	CT-positive (N = 724)	*p* Value
Age	52 (33–66)	48 (30–63)	56 (37–69)	
Sex				
Female	548 (34.5)	318 (36.8)	230 (31.8)	
Male	1,041 (65.5)	547 (63.2)	494 (68.2)	
ASA				
Normal	524 (33.0)	505 (58.4)	395 (54.6)	
Mild disease	900 (56.6)	269 (31.1)	255 (35.2)	
Severe disease	152 (9.6)	84 (9.7)	68 (9.4)	
NA	13 (0.8)	7 (0.8)	6 (0.8)	
Ethnicity				
Asian	19 (1.2)	12 (1.4)	7 (1.0)	
Black	13 (0.8)	10 (1.2)	3 (0.4)	
White	1,531 (96.3)	827 (95.6)	704 (97.2)	
NA	26 (1.6)	16 (1.8)	10 (1.4)	
Psychiatric history				
No	1,369 (86.2)	742 (85.8)	627 (86.6)	
Yes	207 (13.0)	118 (13.6)	89 (12.3)	
NA	13 (0.8)	5 (0.6)	8 (1.1)	
ISS	10 (5–18)	8 (3–13)	16 (9–25)	
Extracranial injury AIS	2 (0–4)	2 (0–3)	2 (0–4)	
Cause of injury				
Incidental fall	751 (47.3)	407 (47.1)	344 (47.5)	
Road traffic incident	588 (37.0)	319 (36.9)	269 (37.2)	
Violence	88 (5.5)	53 (6.1)	35 (4.8)	
Other	138 (8.7)	81 (9.4)	57 (7.9)	
NA	24 (1.5)	5 (0.6)	19 (2.6)	
Pupillary reactivity				
Both reacting	1,499 (94.3)	819 (94.7)	680 (93.9)	
One	22 (1.4)	10 (1.2)	12 (1.7)	
None	9 (0.6)	5 (0.6)	4 (0.6)	
NA	59 (3.7)	31 (3.6)	28 (3.9)	
GCS				
13	92 (5.8)	15 (1.7)	77 (10.6)	
14	275 (17.3)	108 (12.5)	167 (23.1)	
15	1,222 (76.9)	742 (85.8)	480 (66.3)	
Care pathway				
Admission	668 (42.0)	330 (38.2)	338 (46.7)	
ED	523 (32.9)	455 (52.6)	68 (9.4)	
ICU	398 (25.0)	80 (9.2)	318 (43.9)	
PTA				
No	747 (47.0)	477 (55.1)	270 (37.3)	
Yes	714 (44.9)	355 (41.0)	359 (49.6)	
NA	128 (8.1)	33 (3.8)	95 (13.1)	
LOC				
No	620 (39.0)	370 (42.8)	250 (34.5)	
Suspected	209 (13.2)	108 (12.5)	101 (14.0)	
Yes	639 (40.2)	322 (37.2)	317 (43.8)	
NA	121 (7.6)	65 (7.5)	56 (7.7)	
Time to biomarker sampling	11.85 (5.42–18.67)	8.58 (4.25–16.68)	14.83 (7.67–19.81)	
GFAP (ng/mL)	1.41 (0.29–5.51)	0.43 (0.14–1.38)	4.82 (1.88–13.38)	<0.001
NfL (pg/mL)	12.44 (6.89–26.01)	8.73 (5.30–15.85)	20.78 (11.18–44.14)	<0.001
NSE (ng/mL)	14.50 (11.52–20.05)	13.68 (11.04–17.67)	16.48 (12.41–23.57)	<0.001
S100B (ng/mL)	0.11 (0.06–0.20)	0.09 (0.05–0.15)	0.14 (0.08–0.27)	<0.001
Tau (pg/mL)	1.81 (0.98–3.79)	1.28 (0.75–2.20)	3.13 (1.57–6.56)	<0.001
UCH-L1 (pg/mL)	57.06 (27.63–139.88)	37.16 (18.44–72.83)	104.25 (50.02–264.40)	<0.001

Abbreviations: AIS = abbreviated injury score; ASA = American Society of Anesthesiologists; ED = emergency department; GCS = Glasgow coma score; GFAP = glial fibrillary acidic protein; ICU = intensive care unit; ISS = injury severity score; LOC = loss of consciousness; mTBI = mild traumatic brain injury; NfL = neurofilament light; NSE = neuron-specific enolase; MH = medical history; PTA = post-traumatic amnesia; S100B = S100 calcium-binding protein B; UCH-L1 = ubiquitin C-terminal hydrolase L1.

Demographics of the sample: median (interquartile range) for continuous variables; n (%) for categorical. *p* Values comparing the biomarker levels between participants with CT-negative and CT-positive mTBI using the Wilcoxon test with correction for multiple comparisons using the Benjamini-Hochberg method.

### Outcomes

The most common pattern of outcomes was an isolated impaired functional recovery (GOSE score <8), with combined GOSE and PCS impairment being the next most common (eFigure 3). Mental health outcomes (PTSD, anxiety, depression) and SF12v2 MCS scores often occurred in combination and rarely in isolation (eFigure 3). The associations between binary outcome variables are demonstrated in the supplement (eTable 3).

**Figure 3:**
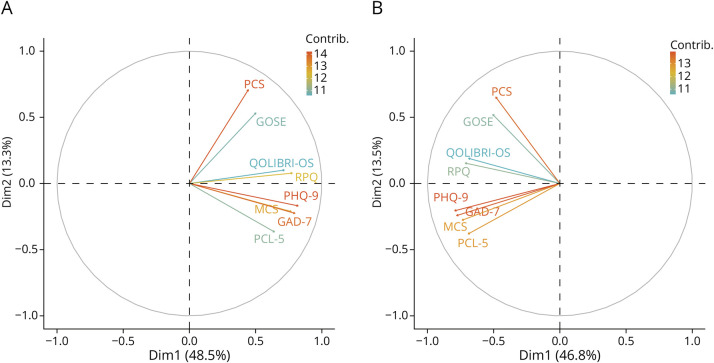
PCA Loading Plot of Binary Outcome Variables in the (A) Participants With CT-Negative Mild TBI With a Complete Outcome Profile (n = 569) and (B) Participants With CT-Positive Mild TBI With a Complete Outcome Profile (n = 523) GAD-7 = Generalized Anxiety Disorder-7; GOSE = Glasgow Outcome Scale Extended; PCL-5 = PTSD Checklist for DSM-5; PHQ-9 = Patient Health Questionnaire-9; QOLIBRI-OS = Quality of Life after Brain Injury-Overall Scale; RPQ = Rivermead Post-Concussion Symptoms Questionnaire; SF12v2 MCS = Short-Form 12-Item Survey version 2 Mental Component Summary; SF12v2 PCS = Short-Form 12-Item Survey version 2 Physical Component Summary; TBI = traumatic brain injury.

PCA of the dichotomized binary outcome variables demonstrated that the first 2 components explained 61.8% of variance in the CT-negative participants and 60.3% in the CT-positive participants (eFigures 4 and 5, eTables 4 and 5). There was separation between the GOSE and PCS measures with the other outcome variables in both participant groups (Figure 2). In the CT-negative participants, the QOLIBRI-OS, SF12v2 MCS, RPQ, and mental health outcomes showed similar trajectories with no clear differentiation pattern. In CT-positive participants, there was more separation, with a grouping of QOLIBRI-OS and RPQ and a separate group of mental health outcomes (PHQ-9, MCS, GAD-7, and PCL-5) (Figure 3).

CT-positive participants had a higher percentage of incomplete recovery (*p* < 0.001, χ^2^ test), PPCS (*p* = 0.033, χ^2^ test), and anxiety (*p* = 0.048, χ^2^ test) compared with CT-negative participants (eTable 6). However, there was no significant difference in relation to impaired disease-specific HRQoL, generic HRQoL, depression, or PTSD (eTable 6).

### Biomarker Associations With Outcomes

#### CT-Negative Participants

On unadjusted comparison, the concentration of all biomarkers, aside from NSE, was significantly raised in participants with a GOSE score <8 compared with those with a GOSE score of 8 (eTable 7). NfL, S100B, tau, and UCH-L1 were significantly raised in participants with SF12v2 PCS impairment (eTable 10). GFAP concentration was significantly lower in participants with QOLIBRI-OS impairment, and GFAP, NSE, tau, and UCH-L1 concentrations were significantly lower in those with sSF12v2 MCS impairment (eTables 8 and 9). GFAP concentration was lower in those with probable anxiety (GAD-7), likely depression (PHQ-9), and probable PTSD (PCL-5), but not significant after adjusting for multiple comparisons (eTables 12–14).

**Figure 4:**
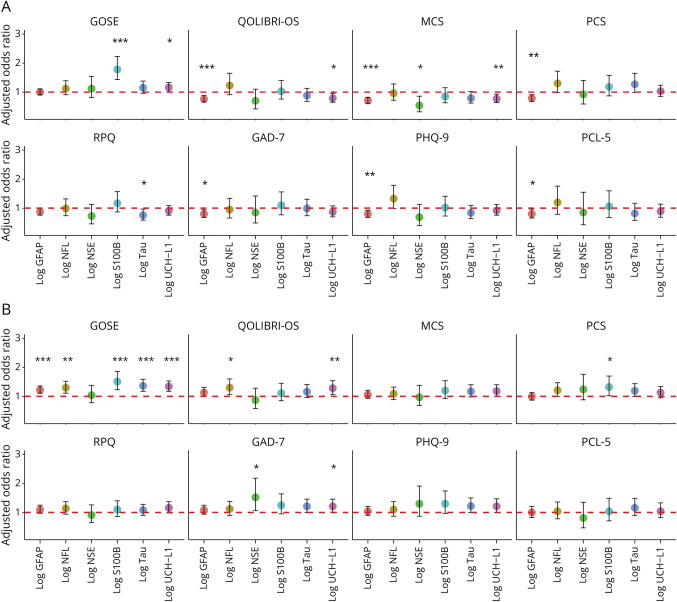
Adjusted Odds Ratios and 95% CIs of Log Biomarkers to Dichotomized Patient Outcomes in the (A) CT-Negative mTBI Cohort and (B) CT-Positive mTBI Cohort Odds ratios adjusted using binary logistic regression for age, sex, extracranial injury, and time to biomarker sampling. ***p ≤ 0.001, **p < 0.01, *p < 0.05 (eTable 15 for details). Panel A showing the results for the C-negative participants and panel B showing the results for the CT-positive participants. GAD-7 = Generalized Anxiety Disorder-7; GFAP = glial fibrillary acidic protein; GOSE = Glasgow Outcome Scale Extended; mTBI = mild traumatic brain injury; NfL = neurofilament light; NSE = neuron-specific enolase; PCL-5 = PTSD Checklist for DSM-5; PHQ-9 = Patient Health Questionnaire-9; QOLIBRI-OS = Quality of Life after Brain Injury-Overall Scale; RPQ = Rivermead Post-Concussion Symptoms Questionnaire; S100B = S100 calcium-binding protein B; SF12v2 MCS = Short-Form 12-Item Survey version 2 Mental Component Summary; SF12v2 PCS = Short-Form 12-Item Survey version 2 Physical Component Summary; UCH-L1 = ubiquitin C-terminal hydrolase L1.

Figure 4 and eTable 15 in the supplement show the adjusted ORs (OR and 95% CI per log unit increase) of the log biomarker concentration to each binary outcome after adjustment for confounders. The model *R*^2^ values were highest for the GOSE (*R*^2^ 0.120–0.158) and SF12v2 PCS (*R*^2^ 0.197–0.218) models, but lower for others, indicating that the biomarker models poorly explained the variance of these outcomes (eTable 15). Higher levels of S100B (OR 1.78, 95% CI 1.43–2.23; *p* < 0.001) and UCH-L1 (OR 1.16, 95% CI 1.01–1.33; *p* = 0.034) were associated with an increased odds of a 6-month GOSE score <8.

Inverse relationships between log biomarker concentrations and impairment were confined to patient-reported outcomes in the CT-negative group and were most consistent for GFAP (Table 2). Increased day-of-injury GFAP was associated with a decreased chance of impairment on the QOLIBRI-OS (OR 0.76, 95% CI 0.66–0.88; *p* < 0.001), SF12v2 MCS (OR 0.71, 95% CI 0.61–0.82; *p* < 0.001), and SF12v2 PCS (OR 0.79, 95% CI 0.68–0.91; *p* = 0.002); occurrence of probable anxiety disorder on the GAD-7 (OR 0.80, 95% CI 0.68–0.95; *p* = 0.010); occurrence of likely depression on the PHQ-9 (OR 0.80, 95% CI 0.68–0.93 per; *p* = 0.005); and occurrence of likely PTSD on the PCL-5 (OR 0.80, 95% CI 0.66–0.97; *p* = 0.028). Other significant inverse associations were observed between UCH-L1 and impairment on the QOLIBRI-OS (OR 0.79, 95% CI 0.65–0.96; *p* = 0.016), UCH-L1 and impairment on the MCS (OR 0.77, 95% CI 0.64–0.93; *p* = 0.006), NSE and impairment on the MCS (OR 0.54, 95% CI 0.32–0.86; *p* = 0.014), and tau and occurrence of possible PPCS on the RPQ (OR 0.76, 95% CI 0.59–0.97; *p* = 0.028).

**Table 2:** Results of Multivariable Logistic Regression of Outcomes to Log GFAP Level in CT-Negative Participants, Demonstrating Negative Associations With HRQoL and Mental Health Outcomes

Outcome	Predictor	Odds ratio	95% CI	*p* Value
GOSE (n = 865)	Log GFAP	1.00	0.91–1.11	0.949
QOLIBRI-OS (n = 611)	Log GFAP	0.76	0.66–0.88	<0.001
MCS (n = 613)	Log GFAP	0.71	0.61–0.82	<0.001
PCS (n = 613)	Log GFAP	0.79	0.68–0.91	0.002
RPQ (n = 605)	Log GFAP	0.88	0.76–1.00	0.056
GAD-7 (n = 585)	Log GFAP	0.80	0.68–0.95	0.010
PHQ-9 (n = 587)	Log GFAP	0.80	0.68–0.93	0.005
PCL-5 (n = 586)	Log GFAP	0.80	0.66–0.97	0.028

Abbreviations: AIS = abbreviated injury score; GAD-7 = Generalized Anxiety Disorder Assessment; GFAP = glial fibrillary acidic protein; GOSE = Glasgow Outcome Scale Extended; HRQoL = health-related quality of life; NfL = neurofilament light; NSE = neuron-specific enolase; PCL-5 = PTSD Checklist for DSM-5; PHQ-9 = Patient Health Questionnaire-9; QOLIBRI-OS = Quality of Life after Brain Injury-Overall Scale; RPQ = Rivermead Post-Concussion Symptoms Questionnaire; S100B = S100 calcium-binding protein B; SF12v2 MCS = Short-Form 12-Item Survey version 2 Mental Component Summary; SF12v2 PCS = Short-Form 12-Item Survey version 2 Physical Component Summary; UCHL1 = ubiquitin C-terminal hydrolase L1.

Results of multivariable logistic regression with adjustment for age, sex, extracranial injury, and time to biomarker sampling. The results for other biomarkers and CT-positive participants are presented in Figures 3 and 4 and eTables 15 and 16.

#### CT-Positive Participants

On unadjusted comparison, the concentration of all biomarkers, aside from NSE, was significantly raised in participants with a GOSE score <8 compared with those with a GOSE score of 8 (eTable 7). NfL was significantly raised in participants with impairment on the QOLIBRI-OS (eTable 8). NfL, S100B, tau, and UCH-L1 were significantly raised in participants with SF12v2 PCS impairment (eTable 10). NSE, S100B, tau, and UCH-L1 were raised in participants with probable anxiety disorder on the GAD-7 (eTable 12).

Where there was CT evidence of brain injury, only positive associations were observed between biomarker concentrations and impairment, robustly so for the GOSE and much less for patient-reported outcomes. Figure 4 and eTable 16 show the adjusted ORs (OR and 95% CI per log unit increase) for each outcome, adjusted for confounders. The model *R*^2^ values were highest for the GOSE (*R*^2^ 0.057–0.09) and SF12v2 PCS (*R*^2^ 0.17–0.18) models. All *R*^2^ values were lower in the CT-positive participant models than in the corresponding model in the CT-negative participants (eTable 16). Higher concentrations of GFAP (OR 1.22, 95% CI 1.11–1.36; *p* < 0.001), NfL (OR 1.30, 95% CI 1.11–1.52; *p* = 0.001), S100B (OR 1.51, 95% CI 1.23–1.86; *p* < 0.001), tau (OR 1.36, 95% CI 1.17–1.59; *p* < 0.001), and UCH-L1 (OR 1.34, 95% CI 1.17–1.53; *p* < 0.001) were associated with increased odds of a GOSE score <8. Further associations were seen between NfL (OR 1.3, 95% CI 1.06–1.60; *p* = 0.011) and UCH-L1 (OR 1.28, 95% CI 1.07–1.54; *p* = 0.009) with impairment on the QOLIBRI-OS, S100B (OR 1.32, 95% CI 1.02–1.70; *p* = 0.033) and impairment on the SF12v2 PCS, and NSE (OR 1.52, 95% CI 1.06–2.18; *p* = 0.021) and UCH-L1 (OR 1.21, 95% CI 1.01–1.46; *p* = 0.041) with occurrence of probable anxiety disorder on the GAD-7.

### Sensitivity Analyses

GFAP levels were lower in participants with a history of treatment of mental health problems, although this was not significant after adjustment for multiple comparisons (eTable 17). The results of both sensitivity analyses are shown in the supplement, with the inverse relationships between GFAP levels and HRQoL/mental health outcomes remaining with (1) the inclusion of psychiatric history (eTable 18) and (2) a subset of patients with symptoms meeting the ACRM criteria for mTBI (eTable 19). Primary GOSE results were replicated in a subset with a complete outcome profile (eTable 20).

## Discussion

This analysis examined day-of-injury serum biomarkers and 6-month patient outcomes. Multivariable analysis showed significant associations between the GOSE scores and biomarkers in participants with CT-negative and CT-positive mTBI, with higher concentrations linked to greater odds of incomplete functional recovery. Most biomarkers did not significantly associate with HRQoL, PPCS, or mental health outcomes in either CT-negative or CT-positive participants. However, in CT-negative participants only, an inverse association was observed between the acute concentrations of GFAP, and, to a lesser extent, tau and UCH-L1, and occurrence of impaired HRQoL or mental health outcomes.

Associations were observed between outcomes in both CT-positive and CT-negative participants, with only the GOSE, and, to a lesser extent, SF12v2 PCS, identifying impairment in isolation from the other assessments. PCA demonstrated a dimension representing function and another representing mental health and well-being, grouping physical HRQoL (SF12v2 PCS) and GOSE scores separate from the mental health outcomes, QOLIBRI-OS and RPQ. This aligns with previous cluster analysis of these outcomes across all TBI severities.^36^ More clearly seen in CT-positive participants, we observed a separate grouping of RPQ and QOLIBRI-OS positioned between the mental health outcomes and GOSE/SF12v2 PCS. These outcome measures contain aspects of both physical and mental symptoms and, as such, may be expected to lie, as was seen in this analysis, between the physical/functional outcomes and the mental outcomes.

Higher concentrations of acute serum biomarkers have been associated with impaired functional recovery in the previous literature,^11,12^ findings supported by our analysis. The GOSE's superior ability to identify impairment after injury compared with other instruments, alongside positive biomarker associations, suggests that it is sensitive to the degree of acute brain injury. It has previously been suggested that the choice of assessment type should be tailored depending on level of functional recovery (GOSE score),^36^ and our findings support this hypothesis.

Although the expectation would be that the severity of intracranial injury will influence patient-reported outcomes, as seen in relation to functional outcomes, we did not observe this. Most of the associations between biomarkers and patient-reported outcomes were not statistically significant in either the unadjusted or adjusted analysis. This demonstrates a lack of relation between the severity of intracranial injury and HRQoL or mental health outcomes after mTBI and indicates that the outcomes are nonspecific to the brain injury and the degree of biological injury sustained. There remains a challenge to reconcile the cause of the differences in acute biomarker expression in those with ongoing functional impairment, where a clear and consistent rise in biomarkers in those with worse functional outcomes has been seen across multiple studies^11,12^ and those seen in relation to patient-reported HRQoL and mental health outcomes, where little association was seen in this study.

On multivariable analysis, a trend of inverse associations was observed in the participants with CT-negative mTBI between GFAP and HRQoL or mental health outcomes. Though seemingly paradoxical, these findings echo previous results observed in different patient cohorts where lower acute levels of GFAP, UCH-L1, and S100B were found in participants who subsequently reported greater levels of postconcussive and PTSD symptoms.^13,14^ Biomarkers, and in particular GFAP, are sensitive for the detection of intracranial pathology on acute CT,^10^ sensitive for the detection of CT occult pathology seen on MRI,^8^ scale to volume of intracranial pathology on acute imaging,^9^ and have utility in the diagnosis of CT-negative mTBI.^37,38^ Therefore, biomarkers (GFAP in particular) may be viewed as a measure of brain injury, both observed or unobserved on the acute CT imaging. A discordant relationship has previously been observed between severity of brain injury and self-reported neuropsychiatric symptoms, with the more mildly brain-injured group reporting the increased severity of neuropsychiatric symptoms.^39^ Furthermore, injury characteristics of a less severe injury have been found to increase the risk of post-traumatic stress symptoms.^40^ The inverse relationships seen between GFAP and HRQoL/mental health outcomes in the participants with CT-negative mTBI are, perhaps, biological reflections of this discordance. Hence, the GFAP, and to a lesser degree, tau and UCH-L1, is raised in participants with greater degrees of unobserved (CT-occult) brain injury, with these participants reporting less mental health and HRQoL impairment.

Different theories have been proposed to explain this discordant relation between injury severity and symptomology.^39^ After identifying a negative association between PTSD and GFAP levels following mTBI in the TRACK-TBI cohort, it was hypothesized that this relationship may partially result from a prolonged duration of post-traumatic amnesia (PTA) in individuals with more severe glial injury.^13^ This may lead to both a higher GFAP and interference with encoding and/or consolidating memory, protecting against PTSD. However, this does not readily explain the negative relationships with other outcomes observed here. Another possible explanation is that participants with CT-negative mTBI were more likely to be discharged from the emergency department, potentially having less access to clinical follow-up and worsening symptoms.^41^ It is also possible that there may be increased cognitive impairment in participants with CT-occult brain damage and higher biomarkers, which may make it more difficult for participants to have the self-awareness to both identify and report worsening HRQoL and symptoms since injury.^42^

Potentially contrary to the abovementioned hypothesis was the marginally greater percentage of participants in the CT-positive mTBI group with impairment on the dichotomized outcomes. The differences, however, were small and only significant in relation to GOSE, RPQ, and GAD-7. This is in keeping with a general lack of association previously observed between mental health and CT abnormalities after mTBI in both a previous CENTER-TBI analysis^7^ and other cohorts.^26^ There are multiple factors aside from injury severity that can differ between participants with CT-negative and CT-positive mTBI that may influence biomarker concentration and/or clinical outcomes, including extracranial injury; ongoing clinical care; and radiologic factors such as intracranial lesion type, volume, location of injury, and subsequent lesion progression.^9^ These factors may mask the more simple associations observed in the participants with CT-negative mTBI and explain the lack of significant associations in the CT-positive participants.

In this analysis, there were higher levels of pre-existing psychiatric disease among the participants with impaired HRQoL and mental health outcomes. There is conflicting evidence concerning the effects of affective disorders on circulating GFAP levels. Histologic study has shown astrocyte dysfunction or density reduction in patients with a history of major depression^43^ while postmortem studies have demonstrated decreases in astrocyte numbers in participants with major depression across multiple brain regions.^44^ Furthermore, an inverse association has been shown between GFAP levels and PTSD symptoms in patients with chronic PTSD.^45^ However, sampling of serum GFAP in participants with depression has demonstrated raised GFAP levels than in controls.^46^ The primary results of this analysis remained unchanged with addition of psychiatric history as an additional potential confounding variable to the logistic regression analysis. However, it should be noted that the documented medical history of psychiatric illness likely serves as an underestimate of the true burden of mental health disorders.

Emotional distress at the time of injury may be an important factor in the observed results. Distressed patients, whether due to traumatic events of the injury or other factors including premorbid anxiety or previous traumatic experiences, may present to health care services with a comparatively lower severity of injury. Significant emotional distress at the time of injury is associated with worse outcomes after TBI.^47^ This population may have a relatively minor injury and, therefore, lower biomarker levels, but an increased risk of poor mental health and HRQoL outcomes. Furthermore, the degree of distress may lower the threshold of the treating clinician for ordering an acute CT scan. CT imaging was a requirement for recruitment to CENTER-TBI, which may introduce selection bias, potentially accounting for the results seen. To address this concern, a sensitivity analysis was performed only including participants who met the ACRM criteria for mTBI.^33^ The results in this subset were consistent with the primary analysis, indicating that the results were not solely driven by selection bias and a cohort of patients without a mTBI. However, overall, the level of acute psychological distress after injury and how this may affect patient presentation and outcomes are poorly characterized in previous TBI studies.^47^ This may represent an important factor in the observed relationships between injury severity, patient phenotype, and patient-reported outcomes in this analysis. Further study of patient outcomes after mTBI, with a greater emphasis on a patients' subjective and objective psychological distress at the time of injury, may potentially offer important insights in this complex picture.

There are several limitations that should be considered in interpretation of the results. Participants were included in the CT-negative mTBI group if they had no findings on CT; however, a proportion of these participants would have CT-occult structural damage that may be visible on MRI.^8^ 24 hours was chosen as the maximal time to biomarker sampling. However, given the heterogeneity in kinetic profiles of the biomarkers, the peak biomarker level may be missed at the actual sampling time. Therefore, time to sampling was included as a covariate in the multivariable analysis. It is important to note that the aim of this study was not to create prediction models for outcomes after TBI but to examine the relation between biomarkers and outcomes. The low goodness-of-fit statistics of the logistic models reflect this. The CIs in the multivariable regression analysis were often wide and close to 1, meaning interpretation of significance should be cautious. Further studies incorporating biomarkers into prediction models, as has been performed in relation to functional outcomes and PPCS,^11,48^ with validation internally or externally, would be required for this purpose. The patient-reported outcomes presented are subject to recall bias, with variability in interpretation and reporting of the instruments. The CENTER-TBI core study cohort is largely White; although broadly reflective of the European population studied, it reduces the transferability of the results to populations with different demographics. Missing data are common in TBI research, and a large number of CENTER-TBI participants were excluded from this analysis.^36^ Overall, the degree of missing outcome data in CENTER-TBI is comparable with other TBI studies^49^ and discussed in greater depth in previous publications.^36^ The primary analysis used a complete case approach for each outcome measure, which can introduce bias if outcome data are missing. The GOSE was the most frequently used outcome measure, leading to a larger sample size. This difference might cause selection bias, potentially causing the variations in biomarker associations between outcome measures. However, a post hoc sensitivity analysis including only participants with complete outcome data replicated the primary results, suggesting that the overall findings are not substantially affected by the bias introduced by missing data. The percentage of CT positivity is higher than might be expected in the clinical mTBI population. In part, this may relate requirement of CT imaging for recruitment in CENTER-TBI and recruitment to 3 separate care pathways. However, the proportion of CT-positive participants (46%) remains greater than observed in the larger CENTER-TBI registry study (n > 20,000, 31% CT-positive), conducted alongside the CENTER-TBI core study to provide “real-world” data for comparison of the representativeness of the core study.^50^ This will partially be due to the higher percentage of participants admitted to ICU in this analysis in comparison with the registry study. However, the high rates of CT positivity present an important limitation when translating the findings of this study to the clinical mTBI population.

Higher concentrations of day-of-injury biomarkers were significantly associated with increased odds of functional impairment in CT-positive and, to a lesser extent, CT-negative mTBI. However, little association was demonstrated between biomarkers and HRQoL/mental health outcomes after mTBI. In CT-negative participants, only the concentration of GFAP and, less consistently, UCH-L1 and tau was inversely associated with impaired HRQoL and the development mental health outcomes at 6 months. Further exploration is required of the patient phenotypes and injury factors that may further explain the complex relationships observed between acute biomarkers and patient-reported outcomes after mTBI.

## Appendix 1 Authors

**Table:**

Name	Location	Contribution
Daniel P. Whitehouse	Perioperative, Acute, Critical Care and Emergency Medicine (PACE), Department of Medicine, University of Cambridge, Addenbrooke's Hospital, United Kingdom	Drafting/revision of the manuscript for content, including medical writing for content; major role in the acquisition of data; study concept or design; analysis or interpretation of data
Lindsay Wilson	Division of Psychology, University of Stirling, United Kingdom	Drafting/revision of the manuscript for content, including medical writing for content; major role in the acquisition of data; study concept or design; analysis or interpretation of data
Endre Czeiter	Department of Neurosurgery, Medical School, and Neurotrauma Research Group, Szentagothai Research Centre, University of Pecs, Hungary	Major role in the acquisition of data
Andras Buki	Department of Neurosurgery, Faculty of Medicine and Health, Örebro University, Sweden	Drafting/revision of the manuscript for content, including medical writing for content; major role in the acquisition of data
Kevin K.W. Wang	Department of Neurobiology, Center for Neurotrauma, Multiomics & Biomarkers (CNMB) Neuroscience Institute, Morehouse School of Medicine (MSM), Atlanta, GA; Program for Neurotrauma, Neuroproteomics and Biomarker Research, Departments of Emergency Medicine, Psychiatry and Neuroscience, University of Florida, McKnight Brain Institute, Gainesville	Drafting/revision of the manuscript for content, including medical writing for content; major role in the acquisition of data
Nicole von Steinbüchel	Institute of Psychology, University of Innsbruck, Austria	Drafting/revision of the manuscript for content, including medical writing for content; major role in the acquisition of data; study concept or design
Marina Zeldovich	Institute of Psychology, University of Innsbruck; Faculty of Psychotherapy Science, Sigmund Freud University, Vienna, Austria	Drafting/revision of the manuscript for content, including medical writing for content; major role in the acquisition of data; study concept or design
Ewout Steyerberg	Department of Biomedical Data Sciences, Leiden University Medical Center, the Netherlands	Drafting/revision of the manuscript for content, including medical writing for content; major role in the acquisition of data
Andrew I.R. Maas	Department of Neurosurgery, Antwerp University Hospital, Edegem; Department of Translational Neuroscience, Faculty of Medicine and Health Science, University of Antwerp, Belgium	Drafting/revision of the manuscript for content, including medical writing for content; major role in the acquisition of data; study concept or design; analysis or interpretation of data
David K. Menon	Perioperative, Acute, Critical Care and Emergency Medicine (PACE), Department of Medicine, University of Cambridge, Addenbrooke's Hospital, United Kingdom	Drafting/revision of the manuscript for content, including medical writing for content; major role in the acquisition of data; study concept or design; analysis or interpretation of data
Virginia F.J. Newcombe	Perioperative, Acute, Critical Care and Emergency Medicine (PACE), Department of Medicine, University of Cambridge, Addenbrooke's Hospital, United Kingdom	Drafting/revision of the manuscript for content, including medical writing for content; major role in the acquisition of data; study concept or design; analysis or interpretation of data

## Appendix 2 Coinvestigators

**Table:**

Coinvestigators are listed at Neurology.org.

## Glossary

ACRM: American Congress of Rehabilitation Medicine
AIS: abbreviated injury scale
CENTER-TBI: Collaborative European NeuroTrauma Effectiveness Research in Traumatic Brain Injury
GAD-7: Generalized Anxiety Disorder-7
GCS: Glasgow coma score
GFAP: glial fibrillary acidic protein
GOSE: Glasgow Outcome Scale Extended
HRQoL: health-related quality of life
INCF: International Neuroinformatics Coordinating Facility
mTBI: mild TBI
NfL: neurofilament light
NSE: neuron-specific enolase
OR: odds ratio
PCA: principal component analysis
PCL-5: PTSD Checklist for DSM-5
PHQ-9: Patient Health Questionnaire-9
PTSD: post-traumatic stress disorder
QOLIBRI-OS: Quality of Life after Brain Injury-Overall Scale
RPQ: Rivermead Post-Concussion Symptoms Questionnaire
S100B: S100 calcium-binding protein B
SF12v2 MCS: Short-Form 12-Item Survey version 2 Mental Component Summary
SF12v2 PCS: Short-Form 12-Item Survey version 2 Physical Component Summary
TBI: traumatic brain injury
UCH-L1: ubiquitin C-terminal hydrolase L1
VIF: variance inflation factor
